# Radiation-induced alterations in histone modification patterns and their potential impact on short-term radiation effects

**DOI:** 10.3389/fonc.2012.00117

**Published:** 2012-09-19

**Authors:** Anna A. Friedl, Belinda Mazurek, Doris M. Seiler

**Affiliations:** ^1^Department of Radiation Oncology, Ludwig-Maximilians-UniversityMunich, Germany; ^2^Clinical Cooperation Group Osteosarcoma, Helmholtz Zentrum München–National Research Center for Environmental HealthNeuherberg, Germany; ^3^Department of Pediatrics, Technical University Munich and Pediatric Oncology CenterMunich, Germany

**Keywords:** post-translational histone modifications, radiation, double-strand breaks, DNA damage response, chromatin

## Abstract

Detection and repair of radiation-induced DNA damage occur in the context of chromatin. An intricate network of mechanisms defines chromatin structure, including DNA methylation, incorporation of histone variants, histone modifications, and chromatin remodeling. In the last years it became clear that the cellular response to radiation-induced DNA damage involves all of these mechanisms. Here we focus on the current knowledge on radiation-induced alterations in post-translational histone modification patterns and their effect on the chromatin accessibility, transcriptional regulation and chromosomal stability.

## Introduction

The genetic information is stored in the DNA, which in eukaryotes is organized in chromosomes. In the first level of DNA packaging, DNA and histone proteins build the nucleosomes where about 147 bp of DNA is wrapped around an octamer of histone proteins. Each two copies of the four main histones, H2A, H2B, H3, and H4, form the nucleosomal core unit. Nucleosomes are linked to their adjacent nucleosome by the linker histone H1. Instead of the canonical histones, some nucleosomes contain histone variants (e.g., H2AX, H3.3, CENP-A) that confer specific functions (Talbert and Henikoff, 2010). The major functions of the canonical histones are DNA packaging and transcriptional regulation. Chromatin structure and function are associated with post-translational modifications (PTMs) of the histone proteins (both canonical and variant), such as acetylation, methylation, phosphorylation, as well as covalent addition of larger groups such as ubiquitin, SUMO (small ubiquitin-like modifier), or poly-(ADP-Ribose). The detection of new PTMs is ongoing (Turner, 2012). PTMs may directly affect the interaction of DNA and histones and thus influence the accessibility of chromatin. For example, it is widely accepted that extensive acetylation of histone tails neutralizes positive charge and thus reduces interaction with negatively charged DNA. There are, however, alternative explanations for the “opening” effect of acetylations (Turner, 2012). The presence of PTMs may increase or reduce binding of other proteins to histone tails and thus affect chromatin structure. It should be noted that direct causality between specific PTMs and a chromatin effect has been demonstrated only for few PTMs (Henikoff and Shilatifard, 2011).

Chromatin structure does not only affect transcription, but all processes requiring access to the DNA, including replication and repair. The paradigm “access-repair-restore,” originally formulated for repair of UV-induced DNA damage (Gong et al., 2005), has also been applied to repair of other damage types, including DNA damage induced by ionizing radiation. Ionizing radiation induces a variety of damage types, among which DNA double-strand breaks (DSBs) are considered as being most relevant for the induction of chromosome rearrangements, cellular survival and long-term genomic stability. Interest in alterations of chromatin structure and PTMs following DSB induction has also been sparked by the fact that a PTM, namely phosphorylation of the histone variant H2AX, is widely used to visualize the chromatin regions surrounding DSBs and also for assessment and quantification of DSB (Dickey et al., 2009). The phosphorylation at serine 139 (S139) of H2AX in response to DSBs is mainly mediated by the kinase ATM (ataxia teleangiectasia mutated), which belongs to a family of phosphatidyl-inositol 3-kinase-related kinases. Two other family members, ATR (ATM- and Rad3 related) and DNA-PK (DNA-dependent protein kinase), can also phosphorylate H2AX. Since phosphorylation occurs in an Mbp-sized region surrounding the DSB, the localization of the DSB and its surrounding chromatin domain can be visualized as so-called foci by immunofluorescence with antibodies recognizing H2AXS139p, which is also termed γ-H2AX (Rogakou et al., 1999). The role of γ-H2AX as platform for the direct and indirect recruitment of a large number of proteins involved in DSB signaling and processing has extensively been reviewed (e.g., Bekker-Jensen and Mailand, 2010; Lukas et al., 2011). For example, by binding to the phosphorylated serine 139 of γ-H2AX, retention of MDC1 in the chromatin domain surrounding the DSB is obtained (Stucki et al., 2005). MDC1 (mediator of DNA damage checkpoint protein 1) is a large mediator/adaptor protein playing a key role in the assembly of radiation-induced foci by its ability to bind various proteins (reviewed by Bekker-Jensen and Mailand, 2010; Jungmichel and Stucki, 2010). These include ATM, NBS1 (nibrin, which is encoded by a gene mutated in Nijmegen Breakage Syndrome) and RNF8 (RING finger protein 8).

So far, γ-H2AX is the best investigated modified histone associated with the cellular response to DSBs, but in recent years analysis of alterations in quantity and localization of other PTMs has gained large interest. Methods to investigate this include analysis of PTM patterns in the γ-H2AX decorated chromatin region by antibody-based immunofluorescence detection and microscopic visualization after DSB induction (e.g., Falk et al., 2007; Solovjeva et al., 2007; Ayoub et al., 2008). Since antibodies detecting histone modifications generally produce a pan-nuclear staining pattern modulated by local alterations in chromatin state, the study of co-localization or mutual exclusion of the PTM in question and γ-H2AX is not easy. While most studies are limited to qualitative assessment of co-localization or mutual exclusion of PTMs, Seiler et al. (2011) introduced methods for quantitative assessment. In addition to DSB induction by ionizing radiation or DSB-inducing chemical agents, such as neocarcinostatin, many IF-based studies used laser microirradiation to investigate PTM patterns at damage sites. A variety of laser microirradiation set-ups have been described to induce, in addition to other DNA damage types, DSBs (Grigaravicius et al., 2009). A disadvantage of the laser-based methods is that the amount and distribution of damage types induced are poorly characterized and that high laser energy densities may lead to unspecific chromatin damage. The high damage load induced by laser-irradiation may also result in microscopically detectable accumulations of proteins that are not found to visibly accumulate after DSB induction with ionizing radiation, not even after irradiation with heavy ions that produce clustered DSBs (for examples see Nagy and Soutoglou, 2009; Splinter et al., 2010; Seiler et al., 2011; Suzuki et al., 2011). Thus, the use of laser irradiation may lead to an over-estimation of response reactions in comparison to more physiological damage situations. An advantage of the targeted irradiation achievable with laser beams is, however, that the site of damage is determined in advance, which facilitates the detection of small alterations and differentiation of irradiated regions from spurious accumulations of damage markers. By using microirradiation with heavy ions, the advantage of localized irradiation can be combined with the production of physiologically relevant damage types (Durante and Friedl, 2011; Seiler et al., 2011).

Chromatin immunoprecipitation (ChIP) offers the possibility of high resolution analysis of chromatin-associated proteins and histone modifications. Its application for the analysis of alterations in chromatin patterns at DSB sites requires site-specific induction of DSBs (e.g., Murr et al., 2006; O'Hagan et al., 2008; Stante et al., 2009; Iacovoni et al., 2010). In general, the size of regions analyzed by ChIP is much smaller than that of regions analyzed by immunofluorescence. In some cases, DSB-associated alterations in PTMs can even be detected by Western blotting of nuclear lysates (e.g., Tjeertes et al., 2009; Seiler et al., 2011), in which case it is assumed that the PTM alterations detected affect also regions not directly adjacent to DSB sites (so-called global alterations). A concern with all antibody-based methods to PTM analysis is potential cross-reactivity. It is, therefore, expected that more specific methods based on mass spectrometry will increasingly be used in future.

Several excellent review articles have recently been published on the topic chromatin and DNA damage response (DDR) (van Attikum and Gasser, 2009; Ball and Yokomori, 2011; Bao, 2011; Xu and Price, 2011; Miller and Jackson, 2012). In the present article, we concentrate on the dynamics of PTMs in response to DSB induction. Over the last years it became clear that not only a large variety of “new” PTMs are formed in the vicinity of DSB sites during the DDR, but that other PTMs appear to be removed from these regions.

## Involvement of PTMs in immediate early damage detection and chromatin opening

Early events of the DSB-induced DDR, starting with ATM-mediated phosphorylation of H2AX, are quite well understood. In contrast, the sequence of immediate early events upstream of ATM is more difficult to elucidate. A major player in recruitment and activation of ATM is the MRN complex, consisting of the proteins MRE11 (meiotic recombination 11), RAD50 (radiation sensitive 50) and NBS1 (for review, see Rupnik et al., 2010). In addition to a function depending on MDC1-mediated recruitment of MRN to the γ-H2AX domain, this complex acts as a DSB sensor, but how exactly it does sense the breaks is not yet clear. Kruhlak et al. (2006) demonstrate a rapid (within 20 s) local expansion of a chromatin region in which DNA damage including DSBs was induced by an UV laser. The expansion did not depend on ATM or H2AX, thus it cannot be explained by events downstream of ATM activation and H2AX phosphorylation. Since this expansion was dependent on ATP, it cannot be solely due to break-induced relaxation of torsional stress. Kruhlak et al. (2006) proposed that a damage sensor mediates decondensation of chromatin.

A candidate for such a sensor is PARP-1 (also known as ARTD1, ADP-ribosyltransferase diphtheria toxin-like 1), the major poly-(ADP-ribose)-polymerase in the cell. PARP-1 can bind to DSBs (and other DNA structures, including single-strand breaks) and is visibly recruited within 1 s to damage induced by laser-microirradiation (Haince et al., 2008). This is accompanied by an extensive poly-(ADP-ribosyl)ation of histones and other chromatin-bound proteins at DSB sites. All core histones, as well as H1, can be subject to poly-(ADP-ribosyl)ation. Recently, in an *in vitro* study Messner et al. (2010) identified the target sites H2AK13, H2BK30, H3K27, H3K37, and H4K16. Whether the same sites are targets of DSB-induced poly-(ADP-ribosyl)ation, remains to be tested. Anyway, *in vitro* and *in vivo*, poly-(ADP-ribosyl)ation of histones leads to increased accessibility of chromatin, which is explained by reduced DNA-histone interaction due to the high density of negative charge in poly-(ADP-ribose) and by recruitment of nucleosome remodeling factors (Messner and Hottiger, 2011; Martinez-Zamudio and Ha, 2012). Unexpectedly, however, after laser-induced damage infliction, Timinszky et al. (2009) observed a higher chromatin density in the damaged region in spite of extensive poly-(ADP-ribosyl)ation, which was due to poly-(ADP-ribose)-dependent recruitment of macroH2A1.1. It is possible that this apparent compaction is preceded by a relative opening of chromatin following poly-(ADP-ribosyl)ation, but further elucidation would require systematic time course experiments.

Besides MRN complex and PARP-1, also the Ku heterodimer consisting of Ku70 and Ku86 has direct DSB end-binding ability. End-binding by the Ku heterodimer and subsequent activation of the catalytic subunit of DNA-PK are required for DSB repair via non-homologous end-joining. Up to now, there is no consistent picture of the interplay between PARP-1, MRN complex and the Ku heterodimer in the initial sensing of DSBs. Binding of Ku has been shown to inhibit binding of PARP-1 and MRE11 (Cheng et al., 2011), while others show an interaction between Ku complex and PARP-1 (Spagnolo et al., 2012). On the other hand, MRE11 binding depends on functional PARP-1 (Haince et al., 2008; Cheng et al., 2011).

Another pathway proposed to explain immediate early chromatin decondensation involves HMGN1 (high mobility group N1)-mediated activation of a histone acetyltransferase, resulting in a global increase of H3K14 acetylation (Lim et al., 2005; Kim et al., 2009). HMGN1, a factor binding to nucleosomes in a constitutive but highly dynamic fashion (Kim et al., 2009), appears to act upstream of ATM in the signaling cascade, since efficient ATM activation requires the presence of HMGN1. However, a role for HMGN1 in rapid local expansion of chromatin after DSB induction has not yet been addressed experimentally.

An impressively large number of ATP-dependent chromatin remodeling factors have been described to accumulate in the vicinity of DSB sites and/or to be involved in the DDR (Lans et al., 2012). Although some of these remodeling factors appear to act at very early steps of the DDR (Ahel et al., 2009; Gottschalk et al., 2009; Lan et al., 2010; Sánchez-Molina et al., 2011), so far no clear candidate has been defined which may be responsible for the rapid decondensation observed by Kruhlak et al. (2006).

In addition to the very early, ATM-independent, chromatin decompaction, also ATM-dependent relaxation mechanisms appear to act after DSB induction (Ziv et al., 2006). These mechanisms involve KAP-1 (Kruppel-associated box domain-associated protein-1), a transcriptional co-repressor involved in DNA condensation. After rapid ATM-dependent S824 phosphorylation of KAP-1 at damage sites, a quick pan-nuclear spreading of the phosphorylated KAP-1 leads to a global increase in nuclease accessibility of the chromatin (Ziv et al., 2006). Global increase in nuclease sensitivity after DNA damage induction has also been observed by others, but its significance is not clear. Since KAP-1 is a barrier to DSB repair in heterochromatin regions, its phosphorylation at S824 serves in addition a more localized role for the repair of DSB located in heterochromatin regions (Goodarzi et al., 2008; Noon et al., 2010) which involves dispersal of the long isoform of CHD3 (chromodomain helicase DNA binding protein 3), one of several possible catalytic subunits of the nucleosome remodeling and deacetylase (NuRD) complex (Goodarzi et al., 2011). Recently it was shown that a second phosphorylation of KAP-1 at S473, which depends on ATM and checkpoint kinase CHK2, promotes mobilization of the heterochromatin stabilizing protein HP1β (Bolderson et al., 2012). It is generally assumed that loss of CHD3 or HP1β facilitates repair in heterochromatin regions by facilitating access for repair factors. Local decondensation may, however, also serve to allow for DSB relocation to regions of lower density via physical forces (Jakob et al., 2011).

Interestingly, damage-associated local chromatin decondensation is not accompanied by a damage-induced localized or global loss of heterochromatin-specific PTMs, such as H3K9me3 or H3K9me2 (Ayoub et al., 2008; Luijsterburg et al., 2009; Sun et al., 2009; Noon et al., 2010; Seiler et al., 2011). It is, however, accompanied by a localized increase of histone H4 acetylation (Murr et al., 2006; Falk et al., 2007; Ikura et al., 2007; Ogiwara et al., 2011), which is mainly conferred by the histone acetyltransferases TIP60 (Tat-interactive protein; Murr et al., 2006) as well as p300 and CBP (CREB-binding protein; Ogiwara et al., 2011). Dispersal of HP1β allows binding of TIP60 which activates its acetyltransferase activity (Sun et al., 2009). DSB-induced hyperacetylation of H4 at lysines 5, 8, 12, and 16 may affect nucleosome stability either directly by reducing the interaction between H2A and H4, or indirectly by involving the NuA4 remodeling complex (reviewed by Xu and Price, 2011). Data on damage-induced hyperacetylation of H3 are conflicting: Ogiwara et al. (2011) observed hyperacetylation of H3K18, which depended on CBP/p300, but not of other N-terminal lysines, whereas H3K14 hyperacetylation was observed by others (Murr et al., 2006; Kim et al., 2009). CBP/p300-dependent hyperacetylation at damage sites has also been described for at H3K56 (Das et al., 2009; Vempati et al., 2010), whereas others observed H3K56 hypoacetylation at damage sites and on a global level (Tjeertes et al., 2009; Yang et al., 2009; Miller et al., 2010, our own unpublished observations). A local decrease of H3K56 acetylation in the vicinity of damage sites would agree well with the observation of local accumulation of histone deacetylases HDAC1 and HDAC2 at damage sites (Miller et al., 2010). However, all results on H3K56 acetylation obtained with antibody-based techniques recently were seriously challenged by Drogaris et al. (2012) due to potential cross-reactivity to other acetylation sites present on H3 N-terminal tails.

Ubiquitination of histones is involved in transcriptional regulation and DDR (Cao and Yan, 2012). The ubiquitin ligase RNF8 directly interacts with MDC1 and is thus recruited to the γ-H2AX domain. RNF8 has in recent years emerged as the starting point of a complex ubiquitin-dependent signaling response (reviewed by Bekker-Jensen and Mailand, 2011; Luijsterburg and van Attikum, 2012) which includes RNF168- and ubiquitin conjugating enzyme UBC13-dependent K63-linked poly-ubiquitination of H2A and H2AX (Huen et al., 2007; Mailand et al., 2007). K63-linked poly-ubiquitination is required for accumulation of downstream repair factors such as BRCA1 (breast cancer protein 1) and 53BP1 (p53 binding protein 1; Lok et al., 2012). Interestingly, after DSB induction RNF168 preferentially targets two novel N-terminal ubiquitination sites (H2AK13/K15) rather than the canonical K119, whereas RNF8 appears to target all three sites (Gatti et al., 2012). RNF8-dependent histone poly-ubiquitination and recruitment of BRCA1 was shown to depend on prior nucleosome destabilization due to H4 hyperacetylation (Ikura et al., 2007; Xu et al., 2010). Others have, however, reported that RNF8- and CHFR (checkpoint with forkhead and ring finger domains)-dependent histone ubiquitination is required for MRG15(MORF4-Related Gene on chromosome 15)-dependent recruitment of the histone acetyltransferases TIP60 and MOF (males absent on the first) to damage sites and subsequent hyperacetylation of H4K16 (Wu et al., 2011), which would place ubiquitination upstream of acetylation. In addition, RNF8 itself appears to have a role in unfolding higher-order chromatin structure which does not rely on its ubiquitin ligase function, but rather on recruitment of the chromatin remodeling factor CHD4 (Luijsterburg et al., 2012).

While recruitment of BRCA1 into foci involves direct interaction of its binding partner RAP80 (receptor-associated protein 80) with the poly-ubiquitin chain (reviewed by Kim and Chen, 2008), the molecular mechanisms of 53BP1 recruitment have long time been enigmatic (FitzGerald et al., 2009; Coster and Goldberg, 2010). 53BP1 binds via its tandem tudor domain to H3K79me1/me2 and/or H4K20me2 (Huyen et al., 2004; Botuyan et al., 2006; Spektor and Rice, 2009), but the damage-specific accumulation of 53BP1 and its dependence on RNF8 cannot solely be explained by damage-induced increase of these methylated sites in the vicinity of DSBs (Huyen et al., 2004; Pei et al., 2011). Interestingly, recent work showed that RNF8 and RNF168 mediate not only the formation of K63-linked poly-ubiquitination chains, but also the formation of K48-linked chains which label the target protein for proteosomal degradation (Meerang et al., 2011; Mallette et al., 2012). RNF8/RNF168-dependent degradation of the histone demethylases JMJD2A (lysine-specific demethylase KDM4A) and JMJD2B (KDM4B) unmasks H4K20me2, thus enabling binding of 53BP1 (Mallette et al., 2012). How these observations reconcile with 53BP1's dependence on K63-linked poly-ubiquitination as described by Lok et al. (2012) remains to be resolved. A similar unmasking mechanism involving the ubiquitin-selective segregase VCP/p95 and chromatin eviction of L3MBTL1 (lethal(3)malignant brain tumor-like protein 1), a chromatin compaction factor, was also described (Acs et al., 2011; Meerang et al., 2011). Interestingly, RNF8 does not only contribute to chromatin opening at damage sites, but may also contribute to the establishment of repressive patterns (see below).

Ubiquitination of H2B is a prime example of participation of mechanisms normally involved in other cellular reactions (in this case transcription) in the DDR. Shiloh et al. (2011) suggest that “borrowing” of factors and mechanisms from other cellular reactions in the case of emergency may help the cell to rapidly respond without having to wait for the synthesis and activation of damage-specific proteins. Mono-ubiquitinated H2BK120 (H2BK123 in *S. cerevisiae*) is associated with transcribed regions of highly expressed genes (Minsky et al., 2008) and mainly found downstream of the transcription start site, hinting at a function in transcriptional elongation rather than initiation. Using chemically defined nucleosome arrays, Fierz et al. (2011) showed that ubH2B interferes with chromatin compaction, leading to an open fiber conformation. The RING finger proteins RNF20 (hBRE1) and RNF40 form the E3 ligase complex responsible for this formation of ubH2B. Recruitment of RNF20/40 to active transcriptions sites appears to be mediated by linking to RNA Polymerase II (RNAPII), e.g., via WAC (WW domain-containing adaptor protein with coiled-coil) protein (Zhang and Yu, 2011). RNF20/40-dependent formation of ubH2B is also seen after induction of DNA damage. Moyal et al. (2011) demonstrate by Western analysis an increase in global levels of ubH2B after treatment with neocarcinostatin, a clastogenic agent, and also a local enrichment of ubH2B at damage sites induced by laser-microirradiation. Since ATM activation and recruitment of early signaling factors MDC1 and RNF8 are not affected by inactivation of RNF20, the authors proposed that RNF20/40 act downstream of signaling, but before initiation of repair. Indeed, recruitment of repair proteins to laser-induced damage sites appears to be reduced if RNF20 is inactivated. Nakamura et al. (2011) similarly demonstrate RNF20 localization to DSB sites, which does not depend on H2AX. They propose that RNF20 accumulation leads to chromatin relaxation, end resection and subsequent recruitment of recombination proteins, such as RAD51 and BRCA1. A role for RNF20/40 in conferring genomic stability was also found by others (Chernikova et al., 2012). So far, it has not been elucidated how RNF20 is recruited to damage sites.

Taken together, several redundant and, at least in part, communicating pathways have been identified which are associated with hyperacetylation, poly(ADP-ribosyl)ation and ubiquitination of histones and may lead to chromatin opening. This raises the question of whether transcriptional regulation is affected by damage-induced alterations in PTM patterns.

## Repressive patterns established in the vicinity of DSB sites

Early evidence that transcription may be inhibited in the vicinity of break sites in spite of the open chromatin configuration came from Solovjeva et al. (2007) who observed that BrUTP incorporation is strongly suppressed at γ-H2AX foci after ionizing irradiation. Similarly, Kruhlak et al. (2007) demonstrated reduced FUrd incorporation in nucleoli microirradiated with a laser beam, suggesting inhibition of RNA polymerase I (RNAPI)-dependent transcription. Transcription inhibition is not a global response, since FUrd incorporation was not affected in neighboring, un-irradiated nucleoli. Inhibition was found to depend on ATM, but not on Ku proteins, JNK (jun N-terminal kinase) pathway or proteasome activity. Shanbhag et al. (2010) developed an elegant system based on induction of site-specific DSBs upstream of a reporter gene to investigate whether the presence of a DSB affects the expression of a (RNAPII-transcribed) gene located *in cis*. By means of fluorescence-tagging, both the nuclease target site (i.e., the location of the DSB) and the nascent RNA of the reporter gene (which contains structures specifically bound by a viral protein) can be visualized. The authors demonstrate a drastically reduced production of nascent reporter transcript upon DSB induction *in cis*, but not a global reduction of transcription. Another similarity of this so-called “DSB-induced silencing *in cis*” to inhibition of RNAPI was that it depended on ATM, but not on the DNA-dependent protein kinase (DNA-PK). In contrast, Pankotai et al. (2012) described dependence on DNA-PK, but not ATM, of DSB-induced transcriptional inactivation of RNAPII-transcribed genes containing target sites for site-specific nucleases. It should be noted that some authors did not observe repressed transcription in the vicinity of break sites (Iacovoni et al., 2010; Cramers et al., 2011). The significance of this discrepancy remains to be elucidated.

Different states of RNA polymerase II (RNAPII) are characterized by different patterns of PTMs in the large subunit, especially in its C-terminal domain (CTD) which consists of 53 copies of a heptapeptide (reviewed by Brookes and Pombo, 2009). Best characterized are the roles of CTD phosphorylation at serine 2 and serine 5. When recruited to the promoter, neither S5 nor S2 are phosphorylated. During initiation, S5 is phosphorylated. The presence of S5 does, however, not necessarily hint at activity of a gene, since paused genes also contain S5 phosphorylated RNAPII. Productive elongation is associated with S2 phosphorylation. To further characterize DSB-induced silencing, Shanbhag et al. (2010) investigated the elongating form of RNAPII. They observed a loss of actively elongating RNAPII with phosphorylated S2 in the vicinity of enzyme-induced DSB sites. At the same time, they did not observe a significant loss of the total amount of RNAPII, as assessed with antibody 8WG16, which recognizes unphosphorylated S2 in CTD repeats. Similarly, a loss of the elongating form, but no reduction in the total amount of RNAPII (in this case measured by an antibody detecting an epitope outside of the CTD), was observed by Seiler et al. (2011) at ion-induced γ-H2AX domains. Chou et al. (2010) and Chagraoui et al. (2011) observed a loss of the elongating form of RNAPII at laser-induced γ-H2AX domains and at γ-H2AX foci induced by UV irradiation after Hoechst 33258-sensitization, respectively. Similarly, underrepresentation of the elongating form of RNA polymerase II is seen in replication-stress-induced so-called OPT (Oct-1, PTF, transcription) domains (Harrigan et al., 2011). OPT domains contain, among other factors, γ-H2AX and 53BP1 and presumably contain damage sites awaiting repair in the next replication phase. At the same time an underrepresentation of the initiating form of RNAPII, which is phosphorylated at S5, was observed by Seiler et al. (2011) and Harrigan et al. (2011). A loss of S5-phosphorylated RNAPII at laser-induced γ-H2AX domains was first described by Miller et al. (2010). Taken together, a picture emerges that transcriptional repression at γ-H2AX domains is associated with a loss of S5- and also S2-phosphorylated RNAPII, but not with a loss of total RNAPII. This is in contrast to mechanisms described for transcriptional inhibition after induction of bulky DNA lesions, such as for example induced by UV irradiation, where RNA polymerase stalls at the damaged site and is then removed by proteosomal degradation after K48-linked ubiquitination (Heine et al., 2008; Hammond-Martel et al., 2012) in a process accompanied by hyperphosphorylation of the CTD, especially at S5. Very recent work (Pankotai et al., 2012) did, however, also imply proteasome-dependent displacement of RNAPII from broken genes after DSB induction.

Ubiquitination of H2A is a well-described PTM associated with transcriptional repression, e.g., in X inactivation, which correlates with ubiquitination of H2AK119 via polycomb repressive complex PRC1 (de Napoles, 2004; Fang et al., 2004). The E3 ubiquitin-protein ligase responsible within PRC1, RING1B (also called RING2 or RNF2), is stimulated by RING1A and BMI-1 (B lymphoma Mo-MLV insertion region 1; Cao et al., 2005), both of which also possess RING finger domains. BMI-1, RING1A, and RING1B are also involved in DSB-associated H2A ubiquitination. BMI-1 is recruited to γ-H2AX domains after site-specific DSB induction, ionizing or laser irradiation (Facchino et al., 2010; Ismail et al., 2010; Chagraoui et al., 2011; Ginjala et al., 2011). Whether it is also recruited to other types of damage, such as UV or hydroxyurea induced damage, is under debate (Ismail et al., 2010; Ginjala et al., 2011). Ismail et al. (2010) report that BMI1 and RING1B are recruited to DSB sites where they confer monoubiquitination of H2AX. BMI1 recruitment did not depend on γ-H2AX or RNF8, but on poly-(ADP-ribosyl)ation at the damage sites and the authors concluded (Ismail et al., 2010; Gieni et al., 2011) that the BMI1-mediated pathway to γ-H2AX ubiquitination acts in parallel to and independent of the RNF8-mediated pathway. In contrast, Ginjala et al. (2011) report that BMI1 recruitment depends on γ-H2AX and RNF8, but not on PARP-1. The work of Ismail et al. (2010) and Ginjala et al. (2011) differs also in the reported effects of inactivation of the BMI1-mediated pathway: while Ginjala et al. did not observe any effect on 53BP1 recruitment, Ismail et al. (2010) reported strong reduction of 53BP1 (as well as BRCA1 and RAP80) foci formation in the absence of BMI1. Chagraoui et al. (2011) reported BMI1 is required for the loss of elongating RNAPII at γ-H2AX domains, thus strengthening the link between recruitment of PRC1 factors and transcriptional repression.

Recent evidence suggests that in addition to PRC1, the polycomb repressive complex 2 (PRC2) is active in the vicinity of damage sites. O'Hagan et al. (2008) observed the appearance of silencing histone modifications, including H3K27me3, in the region surrounding an enzyme-mediated DSB. This was accompanied by the accumulation of several key proteins involved in establishing and maintaining transcriptional repression, including PRC2 core component EZH2 (enhancer of zeste homolog 2), which is the histone methyltransferase responsible for the majority of cellular H3K27me3 marks. Chou et al. (2010) observed recruitment of repressive polycomb complexes at damage sites induced by laser microirradiation, while Seiler et al. (2011) showed EZH2 accumulation at damage sites induced by ion irradiation. Whereas these observations support the involvement of polycomb-mediated silencing (Tang and Greenberg, 2010), no indications for the involvement of heterochromatin-based silencing have been observed. Thus, no DSB-induced increase of the repressive marks H3K9me3 or H3K9me2 could be observed (Ayoub et al., 2008; Luijsterburg et al., 2009; Seiler et al., 2011).

The activity of the polycomb repressive complex PRC2 is inhibited by active chromatin marks, including H3K4me3 (Schmitges et al., 2011). H3K4me3 is a well-characterized active mark primarily associated with the start site of transcription, which at least in part reflects tethering of the COMPASS histone methyltransferase complex to RNA polymerase during active transcription (Henikoff and Shilatifard, 2011; Shilatifard, 2012). H3K4 trimethylation depends also on mono-ubiquitination of H2B. Since ubH2B accumulates at DSB sites (see above), it was suggested that a SET/COMPASS family histone H3K4 methyltransferase may also be involved in DSB repair (Shilatifard, 2012). Indeed, by ChIP analysis of chromatin regions directly flanking nuclease-mediated DSB sites, an increase of H3K4 methylation was seen (Faucher and Wellinger, 2010; Nakamura et al., 2011). In contrast, immunofluorescence analysis after DSB induction by ionizing radiation, coupled with elaborate image analysis methods including ultra-thin sectioning of cells, demonstrated a loss of H3K4me3 and H3K4me2 signals in the γ H2AX domains, which started within minutes after damage infliction and increased over time (Seiler et al., 2011). The loss of H3K4me2/3 signals was associated with a loss of another active mark, H3K9ac, and a loss of active RNAPII. These data are compatible with involvement of JARID1/KDM5 family histone demethylases capable of demethylating H3K4me3 and H3K4me2, presumably as part of complex that also contains histone deacetylases. It is also interesting to note that during ES cell differentiation the PRC2 complex recruits JARID1A (KDM5A/RBP2) to its target genes (Pasini et al., 2008), which couples generation of H3K27me3 with loss of H3K4me3. Recently, depletion of H3K4me3, H3K4me2, and H3K9ac was also observed upon binding of DNA methyltransferase DNMT1 in the context of transcriptional regulation (Clements et al., 2012). Interestingly, this function of DNMT1 was independent of its DNA methyltransferase activity. The authors suggested that depletion of the active histone marks results from DNMT1's interaction with histone deacetylases and demethylases. Since DNMT1 is known to accumulate at laser-induced damage sites (Mortusewicz et al., 2005), it may also serve to recruit histone modifying enzymes in the context of DSB response.

## Conclusion

**Figure 1:**
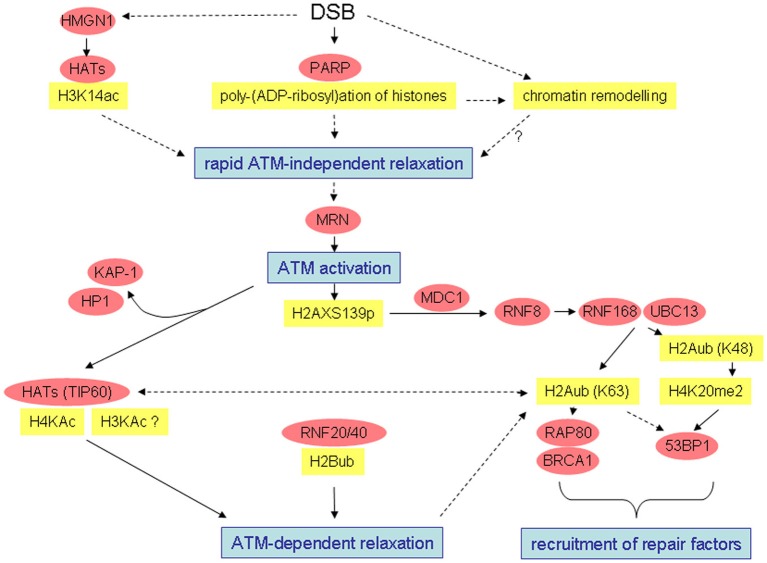
**Simplified overview of radiation-induced alterations possibly leading to open chromatin configuration or chromatin relaxation and recruitment of damage response factors.** Well-characterized interactions and causal relationships are depicted by solid lines, less well-characterized interactions and relationships by broken lines.

**Figure 2:**
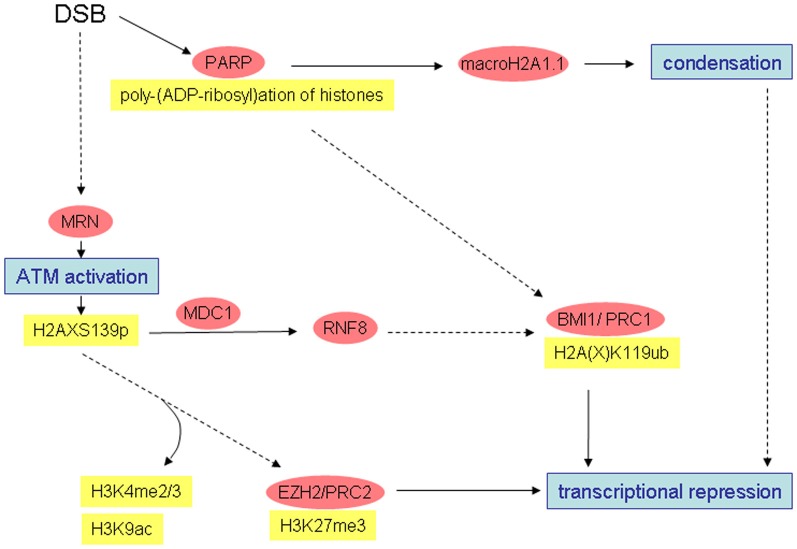
**Simplified overview of radiation-induced alterations possibly leading to chromatin condensation and transcriptional repression.** Well-characterized interactions and causal relationships are depicted by solid lines, less well characterized interactions and relationships by broken lines.

Growing evidence shows that histone modification patterns alter significantly in the course of the cellular response to DSB induction. Both, establishment of patterns suggesting open chromatin configurations and patterns suggesting more condensed configuration were described (see Figures 1 and 2). Many data in the literature are controversial and it is at present not yet possible to reconcile all observations. Observed differences may be explained by different damage types (e.g., clean enzyme-mediated DSBs vs. radiation-induced damage comprising (unclean) DSBs and other damage types), different subcompartments investigated (immediate vicinity of DNA ends vs. γ-H2AX domain) or different time scales. Clearly, more systematic analyses will be required to resolve the open questions.

Any radiation-induced alteration in histone modification pattern has to revert to the original state after successful completion of damage response and repair. The same holds for alterations on other levels of expression regulation such as DNA methylation status or miRNA expression. Otherwise, long-term epigenetic alterations may occur, which then may be causally linked to a carcinogenic process (Mothersill and Seymour, 2003; Loree et al., 2006; Kovalchuk and Baulch, 2008). It will be interesting to determine the relative impact of epigenetic alterations vs. DNA sequence alterations on radiation-induced carcinogenesis.

Finally, since epigenetic alterations contribute to carcinogenesis, the idea of reverting these alterations by so-called epigenetic drugs has lead to the development of several drugs approved for the treatment of cancer (reviewed by Baylin and Jones, 2011). DNA demethylating agents and HDAC inhibitors are generally thought to combat tumour cells by reversing epigenetic silencing of tumor suppressor genes. In the context of the present article, it is interesting to note that these agents posses also radiomodulating activity (reviewed by De Schutter and Nuyts, 2009). Agents leading to DNA demethylation, such as cytidine analogs, possess radiosensitizing activity, at least *in vitro* (e.g., Dote et al., 2005; Brieger et al., 2012; Kim et al., 2012). Radiosensitizing activity of HDAC inhibitors has extensively been studied (reviewed by Camphausen and Tofilon, 2007), but in certain contexts also radioprotective effects were observed (e.g., Konsoula et al., 2011; Miller et al., 2011). Inhibition of poly-(ADP-ribose)-polymerases has emerged as paradigm of synthetic lethal treatment, which is thought to rely on inhibition of SSB repair. During replication, unrepaired SSB will convert into DSB, and in cells deficient in DSB repair via homologous recombination, e.g., breast cancer cells carrying mutations in BRCA1 and BRCA2, cell death will occur (reviewed by Chalmers et al., 2010). It remains to be tested to what extent radiosensitizing effects of PARP inhibition in recombination-proficient cells are caused by inhibition of histone poly-(ADP-ribosyl)ation. The ongoing identification of small molecule inhibitors of histone modifying enzymes opens the way of testing their potential radiomodulating effects in the future.

### Conflict of interest statement

The authors declare that the research was conducted in the absence of any commercial or financial relationships that could be construed as a potential conflict of interest.
